# Model annotation and discovery with the Physiome Model Repository

**DOI:** 10.1186/s12859-019-2987-y

**Published:** 2019-09-06

**Authors:** Dewan M. Sarwar, Reza Kalbasi, John H. Gennari, Brian E. Carlson, Maxwell L. Neal, Bernard de Bono, Koray Atalag, Peter J. Hunter, David P. Nickerson

**Affiliations:** 1 Auckland Bioengineering Institute, University of Auckland, Auckland, New Zealand; 2 Department of Biomedical Informatics and Medical Education, University of Washington, Seattle, Washington, USA; 3 Molecular & Integrative Physiology, University of Michigan, Ann Arbor, Michigan, USA; 4 Center for Global Infectious Disease Research, Seattle Children’s Research Institute, Seattle, Washington, USA

**Keywords:** Epithelial transport, CellML, Physiome Model Repository, Semantic annotation, Model discovery

## Abstract

**Background:**

Mathematics and Phy sics-based simulation models have the potential to help interpret and encapsulate biological phenomena in a computable and reproducible form. Similarly, comprehensive descriptions of such models help to ensure that such models are accessible, discoverable, and reusable. To this end, researchers have developed tools and standards to encode mathematical models of biological systems enabling reproducibility and reuse, tools and guidelines to facilitate semantic description of mathematical models, and repositories in which to archive, share, and discover models. Scientists can leverage these resources to investigate specific questions and hypotheses in a more efficient manner.

**Results:**

We have comprehensively annotated a cohort of models with biological semantics. These annotated models are freely available in the Physiome Model Repository (PMR). To demonstrate the benefits of this approach, we have developed a web-based tool which enables users to discover models relevant to their work, with a particular focus on epithelial transport. Based on a semantic query, this tool will help users discover relevant models, suggesting similar or alternative models that the user may wish to explore or use.

**Conclusion:**

The semantic annotation and the web tool we have developed is a new contribution enabling scientists to discover relevant models in the PMR as candidates for reuse in their own scientific endeavours. This approach demonstrates how semantic web technologies and methodologies can contribute to biomedical and clinical research. The source code and links to the web tool are available at https://github.com/dewancse/model-discovery-tool

## Background

Over the years, computational models of human organ systems have been used to support and improve the diagnosis, treatment and prevention of diseases. These models help scientists investigate specific research questions and hypotheses which may be ethically inappropriate, expensive to study experimentally, or not practically achievable using human or animal subjects. Discovery and exploration of relevant computational models can help scientists more quickly and accurately test their clinical or experimental hypotheses which may involve a broad range of biophysical mechanisms and observations such as disease states, drug actions and vast number of clinical observations. Ultimately this is expected to improve the quality, safety, effectiveness and reduce healthcare costs.

The International Union of Physiological Sciences (IUPS) Physiome Project [1] and the Virtual Physiological Human (VPH) initiative [2] aim at leveraging mathematics and physics-based computational models to represent functional systems of the human body in a computable form, and most importantly, apply our gained physiological insight into clinical practice. In order to achieve this, reproducibility and reuse of computational models are key for building such computational models [3, 4].

A key aspect of the Physiome Project and the VPH is to incorporate semantic annotation of computational models such that these models are accessible, comprehensible, reusable, and discoverable. Here we specifically use semantic annotation to refer to the association of biological knowledge with the relevant constituents of a mathematical model in a computable and reproducible form. Such annotation does not, however, contribute to the mathematical content or interpretation of the model [5]. The biological knowledge added in this way consists of a wide range of contextual information such as species, genes, physiological processes, and anatomical locations. Integration of such information in the computational models requires a diverse set of reference ontologies and often combines concepts from multiple ontologies, i.e. composite annotation [6]. Manual annotation is a time consuming process. We therefore utilize SemGen [7], a semantic annotation tool, to annotate our cohort of exemplar models. To achieve this, SemGen combines concepts from a range of ontologies [8].

In order to discover relevant models, it is essential that models and their semantic annotations, along with other included metadata (e.g. authorship, original publication, etc), are findable and accessible by both people and computational services. In this manuscript, we focus on model discovery within the Physiome Model Repository (PMR) [9], but more broadly, our approach could be applied to a variety of model repositories or resources.

Knowledge discovery is essential within the pharmacology discipline. A recent initiative, Open PHACTS [10, 11], has used semantic querying to enable chemists to discover and explore chemical entities and/or structures. Similarly, other initiatives [12, 13] have developed discovery platforms to explore human diseases and associated genes, as well as other variants using the wealth of semantic resources now available. Pharmacogenomics [14] and biological [15] disciplines heavily depend on knowledge discovery with a view to accessing and integrating web of science resources.

In this paper we present a web-based tool for model discovery that uses the semantic annotations within PMR. Specifically, users can discover models encoded in the CellML format [16], which includes some useful biological information encapsulated within them: species, gene, anatomical location, etc. In the following section, we provide background on the tools and standards we leveraged to develop our web-based model discovery tool. Detailed analysis of the biological information as well as the web tool by which we retrieve this information are discussed in the “Methods” and “Results” sections.

Here we focus on models of epithelial transport as a way of scoping the implementation of our web application. The tools being assembled here into a workflow of model discovery and reuse are, however, of utmost importance when considering the increasingly vast amount of experimental and clinical data being made available. For instance, when considering epithelial Na ^+^ transport, retrieving multiple computational models of the epithelial Na ^+^ channel (ENaC) would be important when considering new experimental data of normal and pathophysiological ENaC channel function since not all channel models may be able to represent the range of function observed. The ability to objectively select appropriate models both semantically and computationally and merge them across multiple scales of physiological complexity will allow researchers to build clinically-useful models that faithfully represent physiological function.

### Tools and standards

Computational tools and standards [17, 18] have evolved over the years to store, exchange and comprehend computational models, including their comprehensive descriptions, i.e. semantic annotation, which make these resources more accessible and discoverable. Some of the software, tools and standards used in our model discovery approach are presented below.

#### RDF and SPARQL

The Resource Description Framework (RDF) is a technology for encoding annotations [19]. RDF is machine readable and allows semantic interoperability between different systems. It represents annotations with a set of triples: subject, predicate, and object. The subject references the element being annotated, the predicate defines the assertion that is being made, and the object is the value being asserted. The COmputational Modeling in BIology NEtwork [20] community has recently recommended the use of RDF for storing annotations on models [21].

The SPARQL Protocol and RDF Query Language (SPARQL) is a query language used to retrieve information from collections of RDF triples [22]. SPARQL and RDF are both widely used semantic web technologies, which together enable “semantic querying”. Our model discovery tool relies extensively on the execution of such semantic queries.

#### CellML

CellML is an eXtensible Markup Language (XML) format that encodes mathematical models of biological phenomena [16]. CellML models are modular and hierarchical, with model authors able to define abstract interfaces to their models by encapsulating complex details beneath a well defined interface. Model components can be reused with an import mechanism allowing external models to be accessible within the importing model. CellML includes the mathematical markup language for representing equations; and makes use of the RDF for the description of metadata such as biological annotations. The CellML metadata specification [23] provides guidelines on how to annotate the biological semantics encapsulated in a CellML model using RDF.

#### Ontologies

For our purpose, we have utilized the Chemical Entities of Biological Interest (ChEBI) ontology [24] to represent solutes such as sodium or hydrogen, the Ontology of Physics for Biology (OPB) [25] to represent biophysical properties such as chemical concentration or fluid flow, and the Foundational Model of Anatomy (FMA) [26] to represent anatomical entities such as the cell nucleus or membrane. By utilizing these knowledge resources, we can create composite annotations that capture, in a computable fashion, specific properties simulated by a model such as concentration of sodium in the collecting duct of the kidney. In addition, we have leveraged the European Bioinformatics Institute’s Ontology Lookup Service (OLS) [27] to retrieve human readable labels from reference ontology URIs.

#### SemGen

SemGen is a toolset for annotating, merging, and decomposing models [7]. It is platform-independent and open source. It is capable of working with models encoded in a variety of modeling formats, including the Systems Biology Markup Language (SBML) [28] and CellML. With SemGen, users can add composite annotations to a model that capture the biological meaning of a model’s contents [6], including the variables in CellML models. These composite annotations link reference terms from knowledge resources such as the OPB, ChEBI, and FMA to form precise, machine-readable descriptions of model variables.

**Fig. 1:**
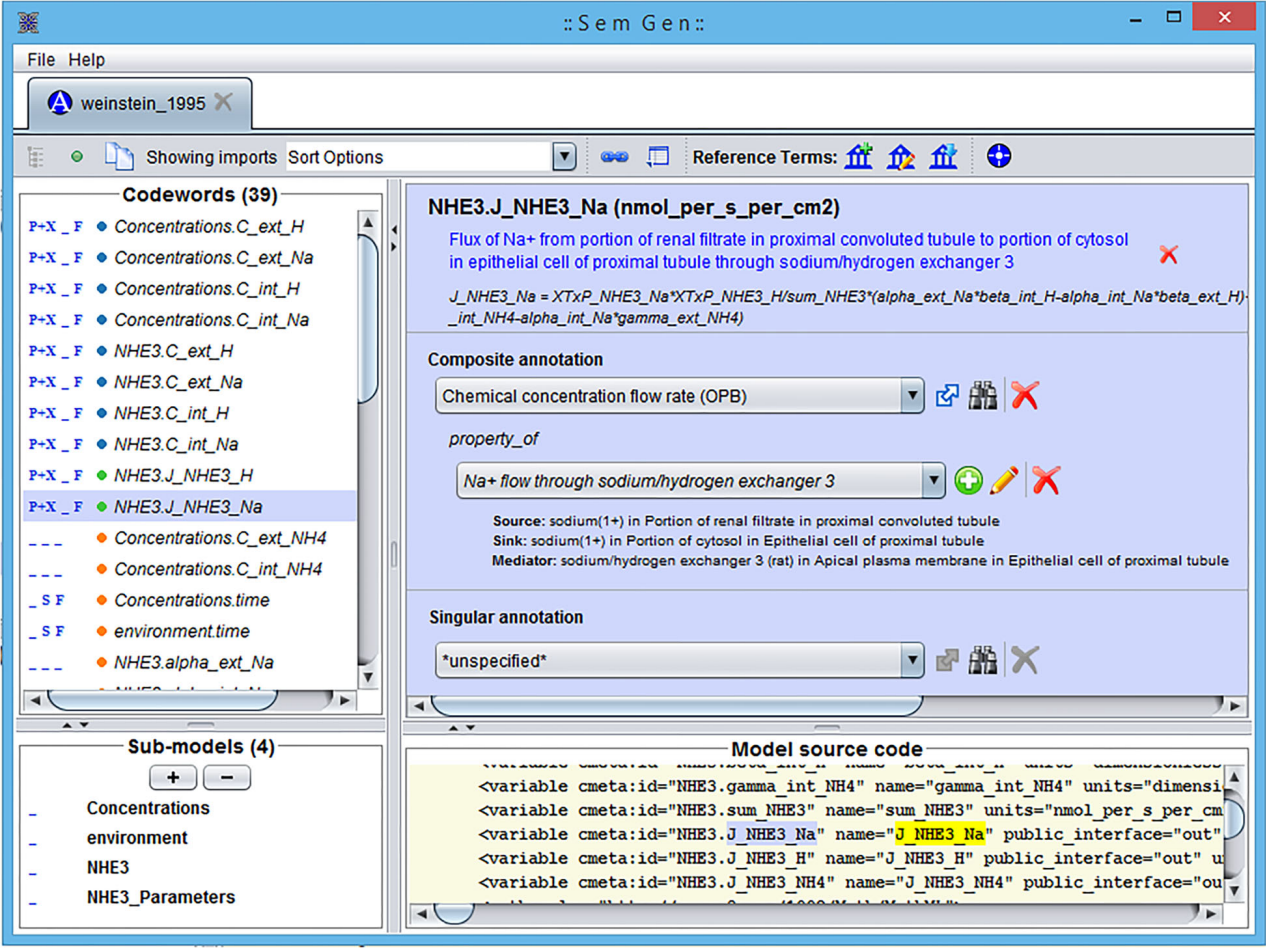
SemGen annotator interface of the sodium/hydrogen exchanger 3 (NHE3) [29] model where codewords identify CellML variables and we have annotated the sodium flux from proximal tubule to epithelial cell cytosol via the exchanger

The SemGen annotator interface of the sodium/hydrogen exchanger 3 (NHE3) [29] model is shown in Fig. 1 wherein the left panel lists 39 CellML variables and 4 CellML components, and the right panel shows a composite annotation for “sodium flux from proximal tubule to epithelial cell cytosol via sodium/hydrogen exchanger 3”, as well as the declaration of that variable in the CellML model code.

#### The Physiome model repository

**Fig. 2:**
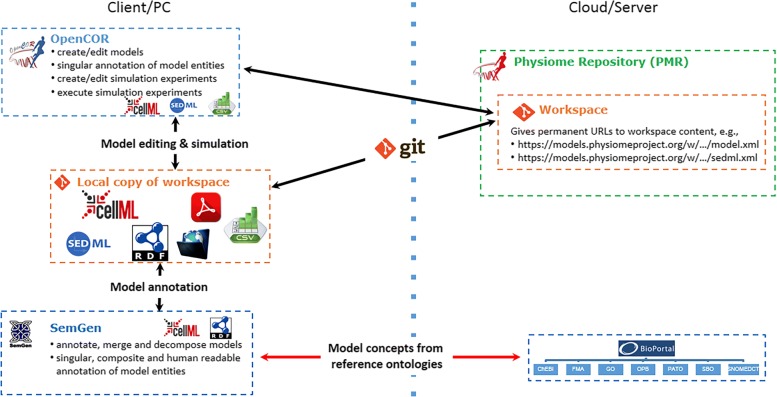
Schematic diagram of synchronization between PMR workspaces as well as OpenCOR and SemGen with the provision of git

The Physiome Model Repository (PMR) was initially designed to store and manage CellML models [9] as part of the IUPS Physiome Project [1]. In the PMR, CellML models can be annotated, exchanged, and reused. PMR also supports a distributed version control system (DVCS) by which users can keep track of the changes made and thereby share and rollback to any committed point. By doing so, it enhances collaboration among model developers. Figure 2 shows an example of synchronizing processes between client and server/cloud applications. In this case, tools like OpenCOR [30] and SemGen maintain local copies of relevant files which are then pulled by the DVCS service (Git).

The PMR has two primary data containers: workspaces and exposures. A workspace is a DVCS repository for a user that contains related information in the PMR. As discussed above, using a DVCS provides the ability to keep track of a number of commit points where users can easily switch to any specific point in the history of a workspace. A workspace can be shared with other users and thereby facilitates collaborative development. A workspace can be synchronized with other compatible DVCS repositories hosted anywhere on the Internet. Private workspaces can be made publicly accessible with the approval of a repository curator. A workspace can be assembled with another workspace, i.e. a hierarchical workspace, which resembles the modularity and reusability features of CellML. One of the key features in a PMR workspace is the indexing of RDF triples into an RDF store so that users can access these triples using SPARQL queries.

Exposures are equivalent to software releases – they provide a specialized view of a workspace at a specific point in the workspace’s history. Users can view the workspace in many ways dependent on the types of content in the workspace. For example, CellML models will have the following views generated: documentation, model curation and metadata, mathematics, source XML, and open with OpenCOR. Exposures are private by default and can be accessible after approval from a repository curator. Exposures can be migrated from one revision of the associated workspace to another as well as a separate “clone” of the workspace that another user might have created when reusing a model.

The PMR also provides web services to address the growing complexity of cross-platform applications though the exchange of messages encapsulated in the HTTP protocol. PMR uses the Open Authorization (OAuth) standard to establish the authentication of a user or consumer and to manage access privileges for services provided by the PMR server. The PMR web services exchange information using the JavaScript Object Notation (JSON) and consumers have to follow specific guidelines [31] to fetch information from a PMR instance. One of the services provided by PMR is a read-only SPARQL endpoint, which can be utilised by application developers to submit SPARQL queries.

## Methods

To demonstrate the value of the semantics-based approaches we propose here, we have developed a platform which allows the user to discover and explore models of interest and then display the annotated information via a web-based interface. We do this in the context of epithelial transport, although as mentioned above these technologies are broadly applicable across the spectrum of biomedical modelling. To provide an initial cohort of models and model entities for use in developing our tool, we first annotated the biological semantics of a set of models in the CellML format using the SemGen tool. These semantic annotations were deposited in PMR to provide resources to discover and assemble on the web interface. Detailed analysis of this process is described below and the model discovery tool can be accessed via https://github.com/dewancse/model-discovery-tool.

### Biological annotation

We annotated a cohort of 33 models on the PMR with biological semantics. This included 12 renal, 11 cardiac, 4 lung, 2 musculoskeletal and 4 miscellaneous epithelial transport models. In particular, we extracted each of the mathematical variables from the models and associated them with biologically meaningful information so that computational modellers and clinicians can discover models relevant to their investigations. These models are all encoded in the CellML format and can be accessed via https://models.physiomeproject.org/workspace/527.

This initial cohort of well-annotated models serves as a proof of concept for testing our implementation. According to the recent community agreement on the harmonization of annotation across the computational modelling in biology community [21], we expect the repository of available annotated models to rapidly grow as the community begins to populate this repository. With community adoption of the harmonized annotation guidelines, we will be able to utilize our platform to query across all available repositories and modelling formats.

**Fig. 3:**
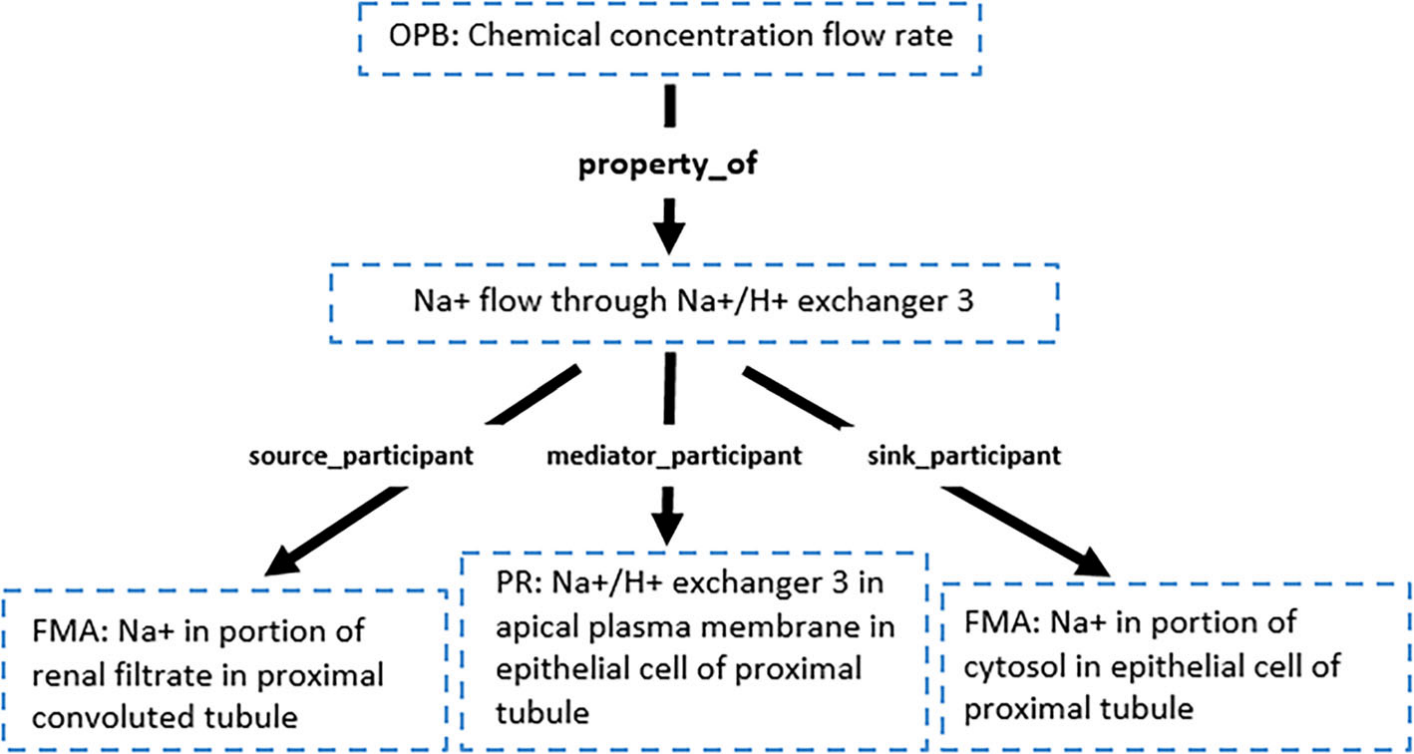
A composite annotation tree for sodium flux from proximal tubule to epithelial cell cytosol via sodium/hydrogen exchanger 3

Resources (variables, components, etc.) in these models can be uniquely identified with URIs composed of the CellML file name (including relative or absolute paths in the PMR) and the document-unique identifiers defined by the CellML format (so-called metadata identifiers). For example, when storing annotations in the same PMR workspace as the NHE3 [29] model, the URI weinstein_1995.cellml#NHE3.J_NHE3_Na unambiguously links to a variable defining the sodium flux through the NHE3 transporter. This example has been annotated in human readable text as “sodium flux from proximal tubule to epithelial cell cytosol via sodium/hydrogen exchanger 3”. Figure 1 shows the SemGen annotator interface for this example annotation formulated as a composite annotation [6]. This demonstration combines concepts from multiple reference ontologies – OPB, FMA and the Protein Ontology (PR). The detailed composite annotation has been presented in Fig. 3.

## Results

In this section we present our web-based interface, Model Discovery Tool, that enables users to discover and explore the annotated biological information in order to investigate specific questions and hypotheses.

**Fig. 4:**
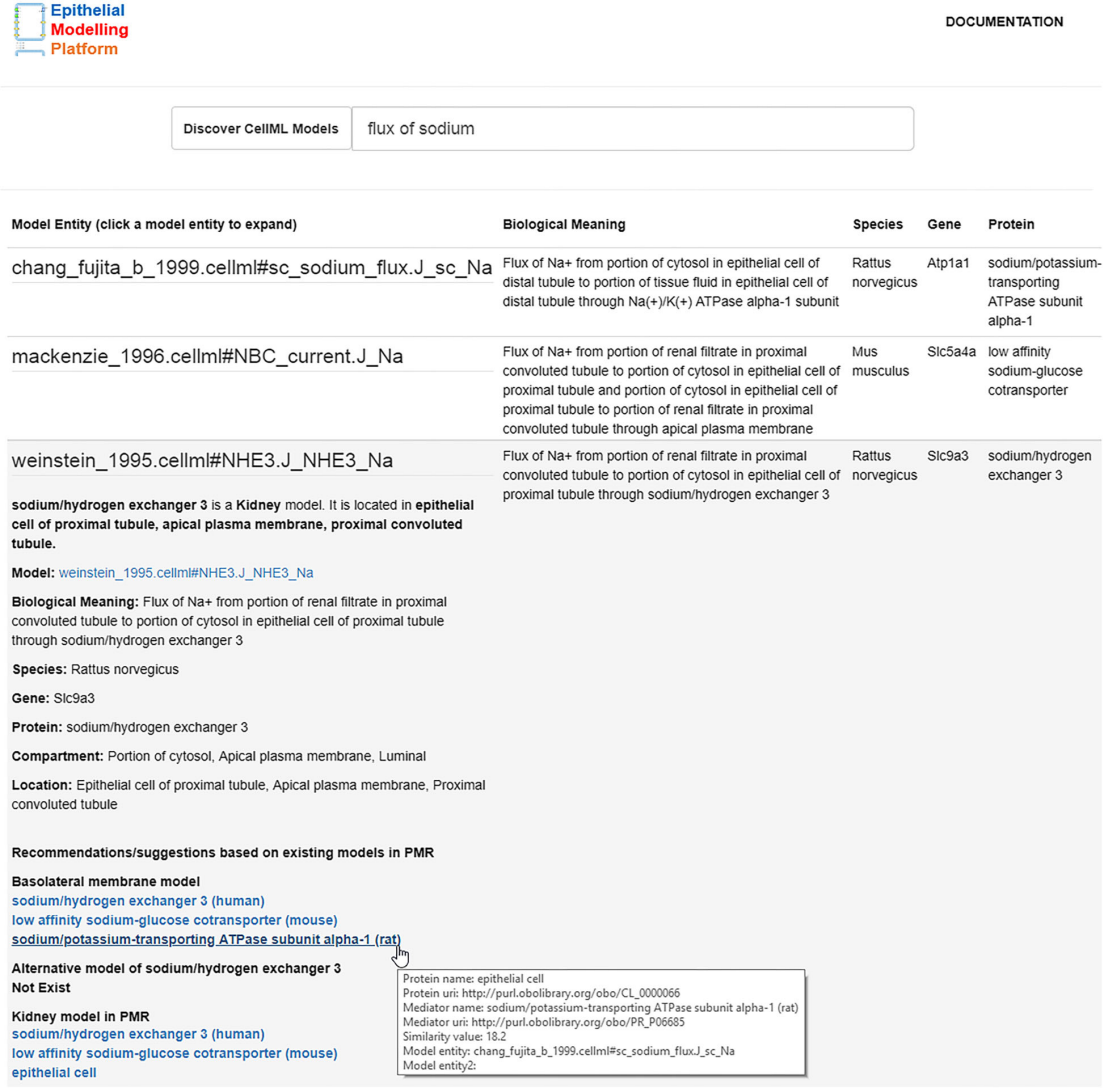
A use case application to search for models in PMR which are relevant to the query *‘flux of sodium’*. For convenience, we have displayed here top three components and the user can scroll down to see other components. By querying the knowledge in PMR this tool retrieves components from the NHE3 model [29], SGLT2 model [32], and the epithelial cell model [33]. Recommendations of models similar to the NHE3 model [29] are displayed as a result of the user selecting the NHE3 sodium flux in the returned results. See text for further details

We have developed a web-based tool to discover and explore models of interest using information extracted from the PMR. Figure 4 shows a list of discovered models for a search query "flux of sodium" from the PMR. From this list, the user can investigate various options such as CellML model entities – model name, component name and variable name, biological meaning deposited in PMR, protein name, and species and genes used during experiments of the associated models.

Users are able to enter human-readable search terms and phrases or choose to enter specific vocabulary terms to use in searches. When discovering resources in PMR relevant to a given search phrase, we first decompose the text into a series of semantic queries which are executed on the PMR knowledgebase and ranked according to how well the discovered resource matches the initial search query. Continuing our example, the phrase shown in Fig. 4 will best match any resource associated directly with a sodium flux, but other sodium-related resources will also be returned with a lower ranking in the search results. To achieve that, we have maintained a static dictionary with key and value pairs to map searched text to reference ontology terms. For example, for a search query “flux of sodium”, the tool will map flux and sodium to OPB and ChEBI ontology terms, respectively, as defined in the dictionary.

In addition, the user can click on a CellML model entity to explore more information as a recommendation or suggestion. As the name implies, this recommendation system suggests for similar or alternative models they users wish to explore or utilize in their model. Machine learning based recommender systems are widely used to build knowledge management systems enabling the user to interact with the system [34–40]. However, our recommender system works in a different manner: it suggests relevant models based on semantic annotations within the PMR and the model the user is currently working with.

Specifically, when the user clicks on a model-entity, the recommender system displays relevant models in the PMR. Initially, it provides a brief overview of the selected model entity: its anatomical location, biological meaning, species, gene, and protein name, as shown in Fig. 4. In this system, we have added a tooltip feature to quickly view some useful information such as protein name and its URI, mediator name and its URI if it is a co-transporter, CellML model entity, and a link to navigate to the associated CellML model.

This information is followed by a list of relevant models in the PMR with respect to the selected model. For example, if the selected model is located in the basolateral membrane, then existing basolateral model(s) are listed as similar models. This populated list is ranked in an attempt to ensure more relevant models are closer to the top of the list.

As discussed above, we focus here on epithelial transport models which are often models of specific membrane-bound transport proteins. To determine which models in the repository are more relevant, we use the WSDbfetch [41] and Clustal Omega [42, 43] services freely available from the European Bioinformatics Institute. For our purpose, we used WSDbfetch to retrieve a protein sequence for a given protein identifier, and the Clustal Omega service to obtain alignment metrics among multiple protein sequences calculated as a similarity matrix.

**Fig. 5:**
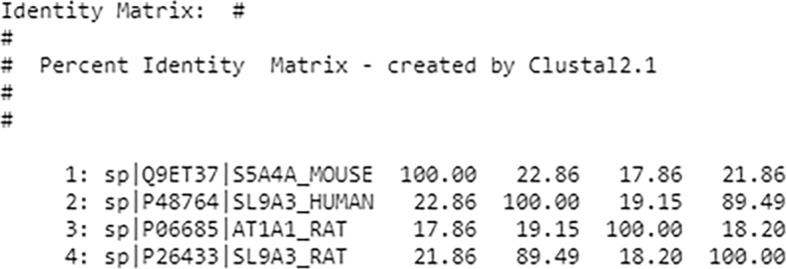
Similarity matrix from the EBI Clustal omega service where protein with UniProt ID P26433 is in the selected model and the rest of the proteins are the basolateral membrane models shown in Fig. 4, (under the "Basolateral membrane model" heading) whose IDs and matrix scores are as follows: P48764 (89.49), Q9ET37 (21.86) and P06685 (18.20)

To get this matrix, we first fetched relevant protein models’ identifier located in the basolateral membrane. Next, we sent these protein identifiers to the WSDbfetch service to retrieve protein sequences. Finally, we sent these protein sequences to the Clustal Omega service to get the similarity matrix. An example of such a similarity matrix is shown in Fig. 5. Along with the biological semantics, the similarity matrix provides a quantitative measure to help rank related models. In this manner, we hope to present the user with the most relevant models first. While these metrics are specific to membrane-bound transport proteins, we envision similar domain-relevant metrics can be developed as our methods are applied within of other domains.

An alternative option shown in Fig. 4 is also provided for the user to explore the same selected model with different species and from various workspaces. The user could also explore relevant organ models and could navigate to the associated workspaces for further investigation.

## Discussion

We have developed a novel semantic annotation and model discovery methodology with the aid of modern tools and standards and implemented a web-based tool build on this methodology. We have made available in PMR an initial cohort of models in the CellML format annotated with biological semantics for use in demonstrating the utility of our methodology and implemented platform.

In our methodology, we make cascading SPARQL calls to explore CellML models in the PMR. These calls first explore model name and biological meaning of a search text string. This text then maps to a static dictionary as key and value pairs with the help of reference ontologies such as the OPB and ChEBI. Subsequent calls fetch protein identifiers associated with a model, and these identifiers are used to retrieve the species and associated gene names for the protein through calls to the OLS. We have also developed a recommender system which leverages semantic annotations in the PMR to identify biologically-similar models.

A limitation of our discovery implementation is that we use a static dictionary with key and value pairs to map searched text to reference ontology terms, i.e. exact matching. However, this could be enhanced with Natural Language Processing (NLP) techniques described previously [44, 45] and by making SPARQL queries to the services provided by the European Bioinformatics Institute. For example, for the text “flux of sodium”, NLP will parse the text into pieces with tokenization, lemmatization, and part of speech techniques and then SPARQL queries could map these pieces onto reference ontology terms. For convenience, the application could also recommend similar words when the user misspells.

## Conclusion

Using semantic annotations to discover models takes into account more contextual information when querying the PMR to provide more relevant results to the user. Using the standard text-based search functionality of PMR, a query for “flux of sodium” returns seven items (5 CellML models and 2 exposure documentation pages). In each case the user is required to navigate to each of those items and determine if they are relevant to their needs. Using our discovery tool, the same query returns over 40 specific entities in our exemplar cohort of CellML models that have been annotated to have some association with “flux of sodium”. These 40 entities, from 12 different models, are ranked so that model variables representing a specific flow of sodium through a cell membrane are nearest the top of the list, and less specific fluxes are nearer the bottom. As shown in Fig. 4, the semantic annotations make it possible to display to the user key information to help them determine relevancy to their work as well as providing recommendations on similar models they may want to investigate.

In addition to the discovery approach, we plan to extend the web application into an Epithelial Modelling Platform that facilitates the reuse and recomposition of epithelial models and their components on the PMR. Our ultimate goal is to create a platform whereby researchers can easily assemble customized models tailored for their specific research needs. The model-discovery tool described here, along with its underlying methodology, represent crucial first steps in the development of such a platform: they offer a solution for identifying and prioritizing modeling components based on a user’s research interests.

## Data Availability

Project name: Model Discovery Tool Project home page: https://github.com/dewancse/model-discovery-tool Operating system(s): Platform independent Programming language: JavaScript, HTML License: Apache 2.0.

## Abbreviations

ChEBI: Chemical entities of biological interest
COMBINE: COmputational Modeling in BIology NEtwork
DVCS: Distributed version control system
ENaC: Epithelial sodium channel
FMA: Foundational Model of Anatomy
IUPS: International Union of Physiological Sciences
JSON: JavaScript object notation
NHE3: Sodium/hydrogen exchanger 3
NLP: Natural language processing
OAuth: Open authorization
OLS: Ontology lookup service
OPB: Ontology of physics for biology
PMR: Physiome model repository
PR: Protein ontology
RDF: Resource description framework
SBML: Systems biology markup language
SPARQL: SPARQL protocol and RDF query language
URI: Uniform resource identifier
VPH: Virtual Physiological Human
XML: eXtensible markup language
